# Does Collocation Inform the Impact of Collaboration?

**DOI:** 10.1371/journal.pone.0014279

**Published:** 2010-12-15

**Authors:** Kyungjoon Lee, John S. Brownstein, Richard G. Mills, Isaac S. Kohane

**Affiliations:** 1 Center for Biomedical Informatics, Harvard Medical School, Boston, Massachusetts, United States of America; 2 Children's Hospital Informatics Program at the Harvard-MIT Division of Health Sciences and Technology, Boston, Massachusetts, United States of America; 3 Operations and Business Affairs, Harvard Medical School, Boston, Massachusetts, United States of America; King's College London, United Kingdom

## Abstract

**Background:**

It has been shown that large interdisciplinary teams working across geography are more likely to be impactful. We asked whether the physical proximity of collaborators remained a strong predictor of the scientific impact of their research as measured by citations of the resulting publications.

**Methodology/Principal Findings:**

Articles published by Harvard investigators from 1993 to 2003 with at least two authors were identified in the domain of biomedical science. Each collaboration was geocoded to the precise three-dimensional location of its authors. Physical distances between any two coauthors were calculated and associated with corresponding citations. Relationship between distance of coauthors and citations for four author relationships (first-last, first-middle, last-middle, and middle-middle) were investigated at different spatial scales. At all sizes of collaborations (from two authors to dozens of authors), geographical proximity between first and last author is highly informative of impact at the microscale (i.e. within building) and beyond. The mean citation for first-last author relationship decreased as the distance between them increased in less than one km range as well as in the three categorized ranges (in the same building, same city, or different city). Such a trend was not seen in other three author relationships.

**Conclusions/Significance:**

Despite the positive impact of emerging communication technologies on scientific research, our results provide striking evidence for the role of physical proximity as a predictor of the impact of collaborations.

## Introduction

As scientific research becomes increasingly complex, investigations frequently involve large-scale collaborations and multi-disciplinary teams [1]. A growing range of communication technologies including email, instant messaging, intranets, wikis, and document sharing systems, are enabling distributed, instantaneous scholarly collaboration irrespective of location. There have been several recent publications on the impact of “big science” and large interdisciplinary teams [2], [3], [4]. The overall message of these publications is that large teams of investigators, especially those working together irrespective of geography (e.g. international consortia) are more likely to be impactful. Understanding the fundamental relationship between collaborator proximity and scientific impact can improve the planning of educational and research facilities and guide the optimal design of large-scale research collaborations.

## Results

**Figure 1:**
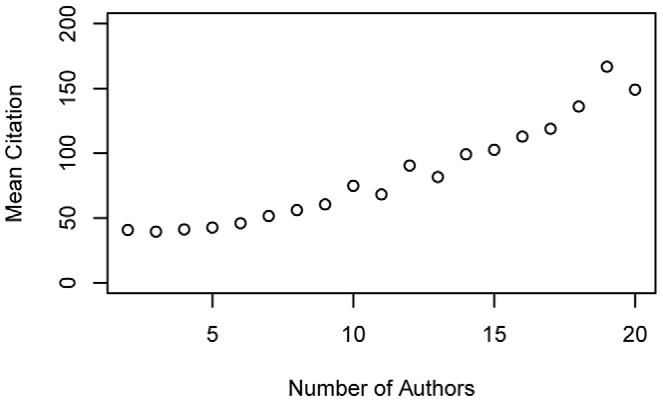
Number of coauthors and mean citations. Citation of an article has strong positive correlation with the number of coauthors. This trend becomes obvious for articles with more than 5 authors. Because of this, to see the relationship between author distance and citation, articles with different number of coauthors need to be analysed separately. We separated articles with 4 or less authors and 5 or more authors.

Figure 1 shows that citation of an article has strong positive correlation with the number of coauthors. This trend becomes obvious for articles with more than 5 authors. Because of this, to see the relationship between author distance and citation, articles with different number of coauthors need to be analysed separately. We separated articles with 4 or less authors and 5 or more authors in the subsequent analysis.

**Figure 2:**
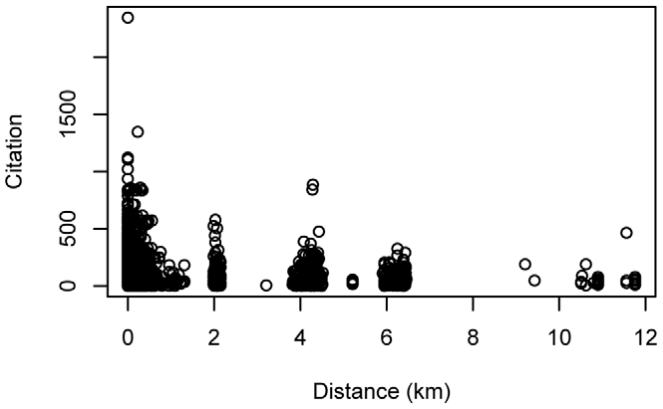
Scatter plot of distance between authors and citation for high resolution data. High resolution data is only available for Harvard affiliated authors. Harvard authors are in 4 major geographical locations: Longwood Medical Area, Massachusetts General Hospital (MGH) main campus, MGH Navy Yard campus, and McLean Hospital. Distances between authors are aggregated in discrete values because authors are not uniformly distributed but in one of those 4 locations. Maximum value (12 km) are for authors in MGH campuses and McLean Hospital.

**Figure 3:**
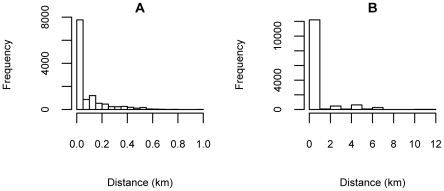
Histogram of data in Figure 2. Most of the coauthors are very close to each other within 200 m.

As Harvard authors are in four major locations (Longwood Medical Area, Massachusetts General Hospital (MGH) main campus, MGH Navy Yard campus, and McLean Hospital), the distance between coauthors are aggregated in discrete values (Figure 2). Maximum values (12 km) between coauthors are for authors in MGH campuses and McLean Hospital. Most of these authors collaborated with people within 200 m (Figure 3).

**Figure 4:**
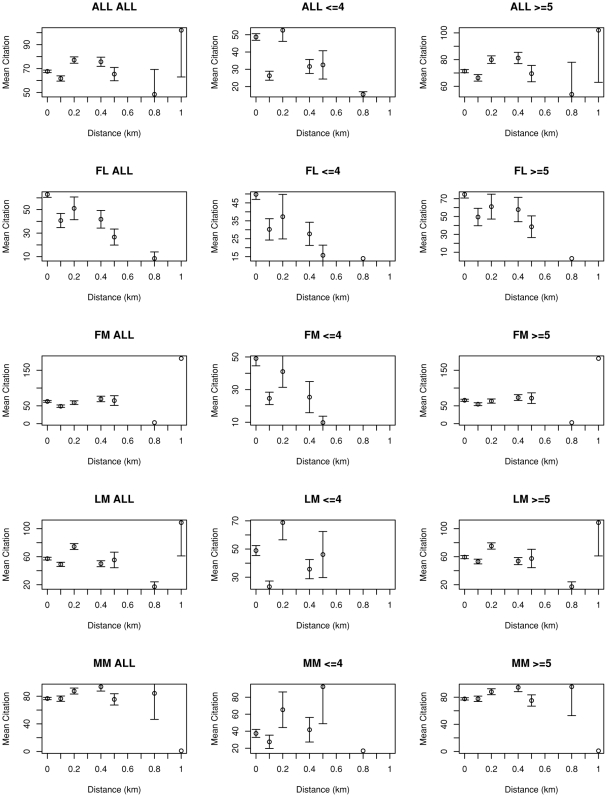
Distance and mean citation in different author relationships. There are four author relationships within an article: first-last, first-middle, last-middle, middle-middle. While there is only one FL in any article, there are (n-2) FMs or LMs in an article with n authors, and (n-2)x(n- 3)/2 MMs. As you can see in FM/LM/MM graphs, the ALL graphs are dominated by > = 5 data because there are way more same relationship pairs as n increases. If there is an article with 100 authors/50 citations, for example, the citation 50 gets counted 98 times in FM/LM mean citation and ∼10000 times in MM. There is one safe case, though. If there are 4 authors, there is only 1 MM. If there are less than 4 authors, there is no MM. Therefore, graphs for FL and MM < = 4 are safe for interpretation. Other graphs should be interpreted carefully.

Figure 4 shows coauthor distance and mean citation for all four author relationships within an article: first-last (FL), first-middle (FM), last-middle (LM), middle-middle (MM). While there is only one FL in any article, there are (n-2) FMs or LMs in an article with n authors, and (n-2)×(n-3)/2 MMs. FM/LM/MM graphs show that the ALL graphs are dominated by > = 5 data because there are many more same relationship pairs as n increases. If there is an article with 100 coauthors and 50 citations, for example, the citation 50 gets counted 98 times in FM/LM mean citation and ∼10000 times in MM. There is one safe case, though. If there are 4 authors, there is only 1 MM. If there are less than 4 authors, there is no MM. Therefore, graphs for FL and MM< = 4 are safe for interpretation. Other graphs should be interpreted carefully.

**Figure 5:**
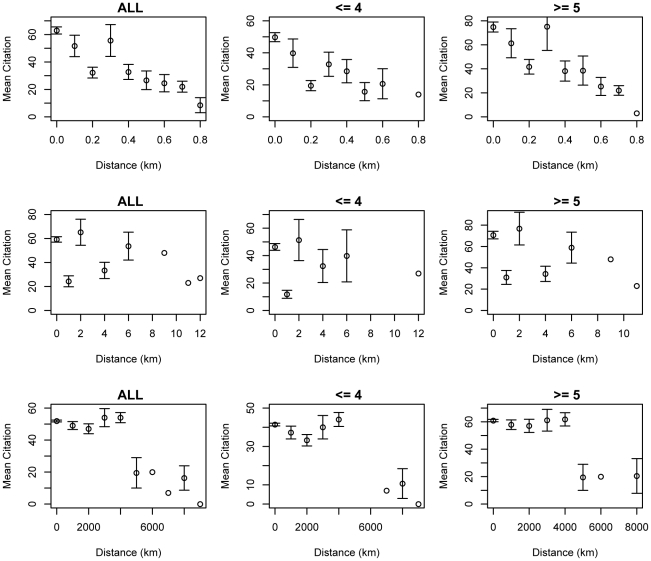
Distance and mean citation for first-last author relationship in three resolutions (100 m, 1 km, and 1000 km).

**Figure 6:**
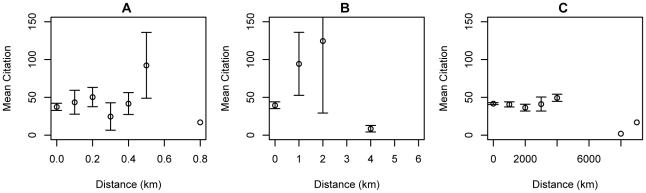
Distance and mean citation for middle-middle author relationship in articles with less than 5 coauthors in three resolutions (100 m, 1 km, and 1000 km). There is no obvious trend.

**Figure 7:**
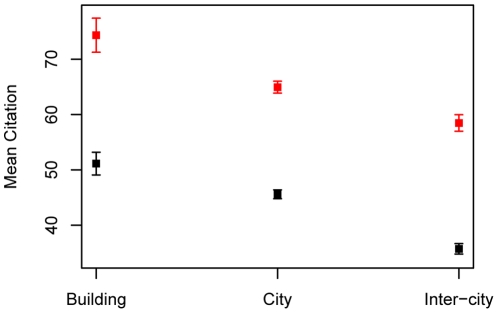
Mean citation for first-last authors in the same building, same city, or different city. Scatter plot showing relationship between first author-last author distance and publication citation impact (±2 SEM). Three inter-author distances were selected for illustration: same building, same city or different cities. Results are plotted for publications with four or fewer authors (black), and with five or more authors (red).

**Figure 8:**
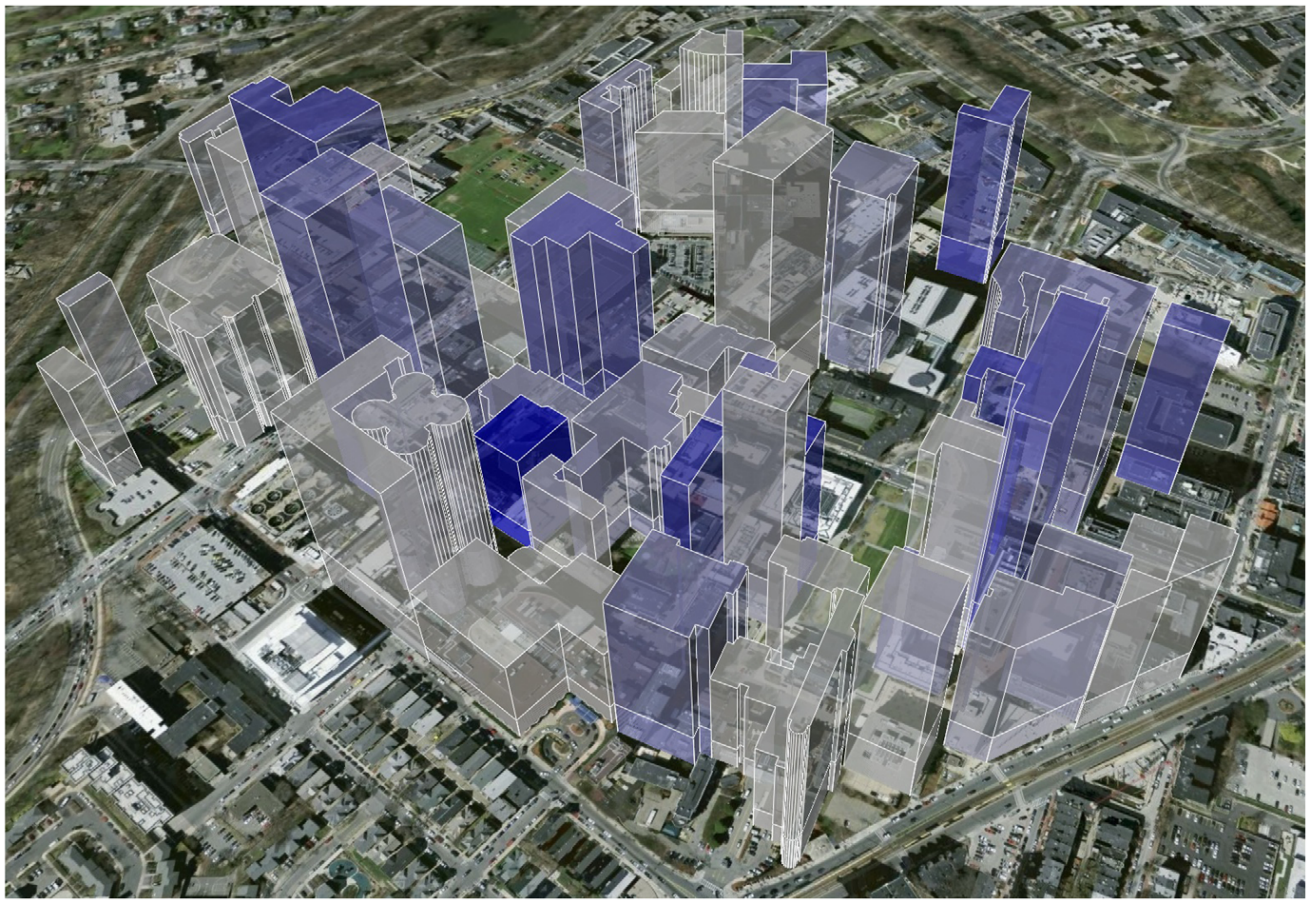
3-D representation of the relationship between intra-building collaboration and mean citation impact on the Longwood campus of Harvard Medical School. The height of each building reflects the mean number of citations of publications originating in that building, and the color gradient reflects the proportion of publications originating from that building in which both first and last authors work in the building (from grey = low to blue = high). An interactive version of this map could be found at http://collaboration.harvard.edu.

Figures 5A and 7 show significant relationships between proximity and impact: the closer the first and last author, the greater the number of citations, an effect that is not evident between middle authors (Figure 6). Figure 8 shows that buildings with a higher-proportion of publications resulting from intra-building collaborations (as opposed to inter-building collaborations) also tend to have higher mean citations among all publications with at least one author working in the building (ρ = 0.50, n = 87, p-value  = 1.0×10−6 by Spearman's rank correlation test).

## Discussion

The results of this first-of-a-kind study suggest that although emerging communication technologies have radically transformed the style and scope of collaboration around the world, physical proximity continues to play a critical role in predicting the impact of scientific research. Although causal relationships cannot be inferred from observational data, a few important associations can be identified. First physical proximity of collaborators was found to be positively associated with publication impact. This effect is most notable for proximity between first and last authors and was not found for other author combinations. Second, the level of intra-building collaboration is positively associated with the impact of publications originating in that building.

There are a number of possible explanations for these associations. It may be that physical proximity truly allows for better collaboration, resulting in higher quality research that tends to be cited more often. It may also be that investigators have a strategic preference for keeping potentially high impact projects wholly within their own laboratory or close circle of research associates.

There have been numerous articles [5] that reported Open Access publications have higher chance to be cited more. It may be that publications in Open Access journals have higher citation, which may not necessarily be related to collaboration and collocation. However, its impact on our results is uncertain as there are also growing number of articles that are reporting no evidence of Open Access advantage [6], [7] in different disciplines.

Previous studies [4] have found that publications arising from international collaborations are associated with greater citation impact than those arising from local collaborations. In this study, we examined the effect of pair-wise inter-collaborator distance within a local framework and found that impact increases with proximity. Therefore advising or make an institutional policy to do international collaboration may not just work at the individual level. It might be more effective to guide faculty to arrange space so that there are more direct interaction with the students and postdocs.

Further work is needed to more deeply understand these relationships and to validate these results across multiple institutions, historical periods, fields of study and measures of scientific productivity. It is possible that these results are only valid for institutions that have similar organizational structure as Harvard University where most of the faculties are appointed to Harvard Medical School as well as one of the totally independent affiliated hospitals. It may be that this unique organizational structure defined the scientific sub-community, which is reflected as collocation.

It is also possible that these results are unique to biomedical sciences. Therefore, it would be necessary to validate these results using other bibliographic databases in other discipline such as the Association for Computing Machinery's (ACM) Digital Library and IEEE's Xplore Digital Library.

## Materials and Methods

As part of our CoCo (collocation-collaboration) project, we analyzed the relationship between collaborator proximity and scientific impact as measured by publication citations. We assembled an unprecedented high-resolution geographic record of scientific collaboration based on the individual offices and laboratories of researchers in a large academic centre.

We focused on life sciences research across three major Harvard University campuses: the Faculty of Arts and Sciences campus in Cambridge, the Longwood campus of Harvard Medical School and the Cambridge and Boston campuses of Massachusetts General Hospital. We analyzed all PubMed-indexed publications with at least one Harvard author published in the years 1999 through 2003, for a total of 35,000 articles across 2,000 journals by 200,000 authors. Since author affiliations were only listed at the institutional level, we performed detailed geo-historical investigation to identify and pinpoint the three-dimensional office location of each author in each specific year.

To study the effect of collaboration size, we divided the publications into those with four or fewer authors and those with five or more. Because of the traditionally privileged roles attributed to first and last authors in the biomedical literature, we highlighted the effect of distance particularly for these author positions and compared them to middle authors.
